# Evaluation of the Italian version of the elderly mobility scale in older hospitalized patients

**DOI:** 10.3389/fpubh.2023.1274047

**Published:** 2023-11-15

**Authors:** Moreno Nicolai, Elisa Casoni, Emanuela Bertino, Letizia David, Chiara Polverigiani, Federica Mallucci, Paola Fioretti, Sara Leonzi, Roberta Bevilacqua, Federico Barbarossa, Elvira Maranesi, Marco Baccini, Ilaria Barboni, Giovanni R. Riccardi

**Affiliations:** ^1^Rehabilitation Unit, IRCCS INRCA, Ancona, Italy; ^2^Scientific Direction, IRCCS INRCA, Ancona, Italy; ^3^IRCCS Fondazione Don Gnocchi, Firenze, Italy

**Keywords:** older people, validation, elderly mobility scale, Italian version, hospitalized patients

## Abstract

**Introduction:**

Reliable and valid assessment tools are needed to evaluate and predict physical function in older hospitalized patients. The aim of this study is to develop the Italian version of the Elderly Mobility Scale (I-EMS) and to evaluate its validity and inter-rater reliability for use with geriatric inpatients.

**Methods:**

The study consists of two phases: (i) translation, where EMS version 2 was translated into Italian by two teams, each comprising 2 bilingual physiotherapists; translations were back translated by 2 native English speakers, and a committee of 2 physiotherapists and 1 physician refined the initial I-EMS version, which was pilot-tested for clarity in a group of ten experienced geriatric physiotherapists; (ii) an observational study assessed I-EMS metrics (reliability, validity) in older Italian inpatients at IRCCS INRCA (Ancona, Italy) between September 2022 and April 2023.

**Results:**

No statistically significant differences were found between the scores of individual items and the total score assigned by different raters. The ICC for total I-EMS was 0.951, SEM was 1.10 and MDC95 was 3.06. The absolute agreement and weighted kappa for individual items ranged 80.32–100% and 8.2–1, respectively. The validity of I-EMS was supported by a significant (*p* < 0.05) correlation with the Barthel Index (*r* = 0.827 and 0.834 for the I-EMS administered by rater A and rater B, respectively).

**Conclusion:**

I-EMS showed good internal consistency and inter-rater reliability, and confirmed construct validity with respect to BI. Therefore, it can safely be used as an assessment tool for hospitalized Italian geriatric patients.

**Clinical trial registration:**

[ClinicalTrials.gov], [NCT05806242].

## 1. Introduction

Population aging is a global issue. Every country in the world is experiencing growth in both the size and the proportion of older people in the population. The World Health Organization (WHO) estimates that the share of population aged 60 years and over will increase from 1 billion in 2020 to 1.4 billion in 2030, so that 1/6 of world population will be 60 years old and over. By 2050, people aged 60 years and older will double (2.1 billion) representing 26% of global population. Similarly, the number of people aged 80 years or older is expected to triple between 2020 and 2050 to reach 426 million (1). With the increase in non-communicable diseases in old age (2), the prevalence of frailty and functional dependence is also expected to increase in the world population. The current situation and future prospect for Italy are similar to those of the world, with people over 65 years old currently representing 23.5% and reaching 34.9% in 2050 of the entire national population (3). In this scenario, current and future health needs are expected to reflect the demographic changes, so specific tools for the clinical and functional assessment of the older patient will be increasingly needed. Many validated and internationally recognized tools for functional assessment are available. The most widespread are Barthel Index (BI) and Functional Independence Measure (FIM), which are not age-specific. The Rivermead Mobility Index (RMI) and the Modified Rivermead Mobility Index (MRMI) also allow functional mobility assessment by observation while the patient performs a series of increasingly difficult motor tasks, but they are both specific for patients suffering from neurological conditions or limb loss and amputation (only RMI). Similarly, the Short Physical Performance Battery (SPPB) allows the clinician to assess functional mobility, but it requires patient’s ability to stand independently, so it is often inadequate for evaluation of frail older patients in acute care setting. By contrast, the Elderly Mobility Scale (EMS) is available as an objective assessment measure of mobility and motor function in older adults (4). It was designed in 1994 as a specific performance measure for frail older patients in acute care setting, reliable and valid for inpatient aged 55 and over. The EMS is composed of 7 items evaluating several complex motor tasks according to a hierarchical sequence (5). Total score is the arithmetic sum of individual scores and ranges from 0 (totally dependent) to 20 (independent mobility).

Validity of EMS has been largely demonstrated by comparison with FIM, Barthel Index (4, 6) and Modified Rivermead Mobility Index (7). It showed excellent inter-rater reliability regardless of physiotherapist’s level of clinical experience and good intra-rater reliability (8). Moreover, it is a handy tool, consuming about 5–10 min for patient evaluation (8) and requiring no specific training for the administration; only previous familiarization with tool is recommended (6). Compared to other assessment measure, the EMS can be easily used for patients who are unable to stand or move from bed (5), resulting an ideal tool for evaluation of frail older patients in acute care setting. The EMS may also be used to analyze the effect of rehabilitation, with a Minimal Clinically Important Difference (MCID) of 2 points or 10% of scale width (5). Compared with BI and Functional Ambulation Category (FAC), the EMS has been shown to be more likely to identify improvement in mobility and the magnitude of detected improvement was greater (6). Moreover, a significant association between low EMS score and personal history of 2 or more falls is documented (8).

Although many factors contribute to determining the discharge destination of frail older inpatients (5, 9) such as functional independence, cognitive status, housing conditions and family support, EMS can help the clinician in the decision. In particular, the final score of EMS showed the ability to divide patients into 3 categories: dischargeable to home (EMS ≥ 14), dischargeable to home with a caregiver (EMS between 6 and 13) and dischargeable to a nursing home (EMS < 6) (5).

Validation studies of the Norwegian EMS (N-EMS) (10), the Swedish Modified EMS (Swe M-EMS) (11) and the Dutch EMS (12) are currently available. In relation to the knowledge of the authors, the Italian version of EMS is not currently available. The aim of this study is to offer an Italian version of the Elderly Mobility Scale (I-EMS) and to evaluate its validity and inter-rater reliability for use with geriatric in-patients. We have chosen to translate the Version 2 of the EMS, corrected by Smith (1994a) (13, 14), with the revised Functional Reach measurement section. We suggest that Barthel Index (15) is a suitable tool for determining construct validity (hypothesis testing) of I-EMS, similarly to what was done for the validation of the original version (4, 16). As well as EMS, the Barthel Index, including even complex tasks, evaluates the patient’s level of autonomy regardless of the underlying disease and allows patients to be stratified on the basis of assistance needs. Furthermore, the Barthel Index is an easy-to-use tool that does not require special training and is easy to apply even for frail older patients in an acute care setting.

The aim of this study is to develop the Italian version of the Elderly Mobility Scale (I-EMS) and to evaluate its validity and inter-rater reliability for use with geriatric inpatients.

## 2. Materials and methods

This study was an observational validation study, aimed to test the validity of the I-EMS. The study was divided into two separate phases: (i) translation phase, and (ii) field test of the I-EMS in order to examine its metric properties (reliability and validity) in the Italian older in-patient population. The study was approved by the Ethic Committee of the IRCCS INRCA Hospital (Ancona, Italy) (process No. 43919/2021) and was registered on ClinicalTrials.gov with trial registration number NCT05806242 (10 April 2022). All the participants were given written information and signed an informed consent.

### 2.1. Phase 1: translation

**Figure 1:**
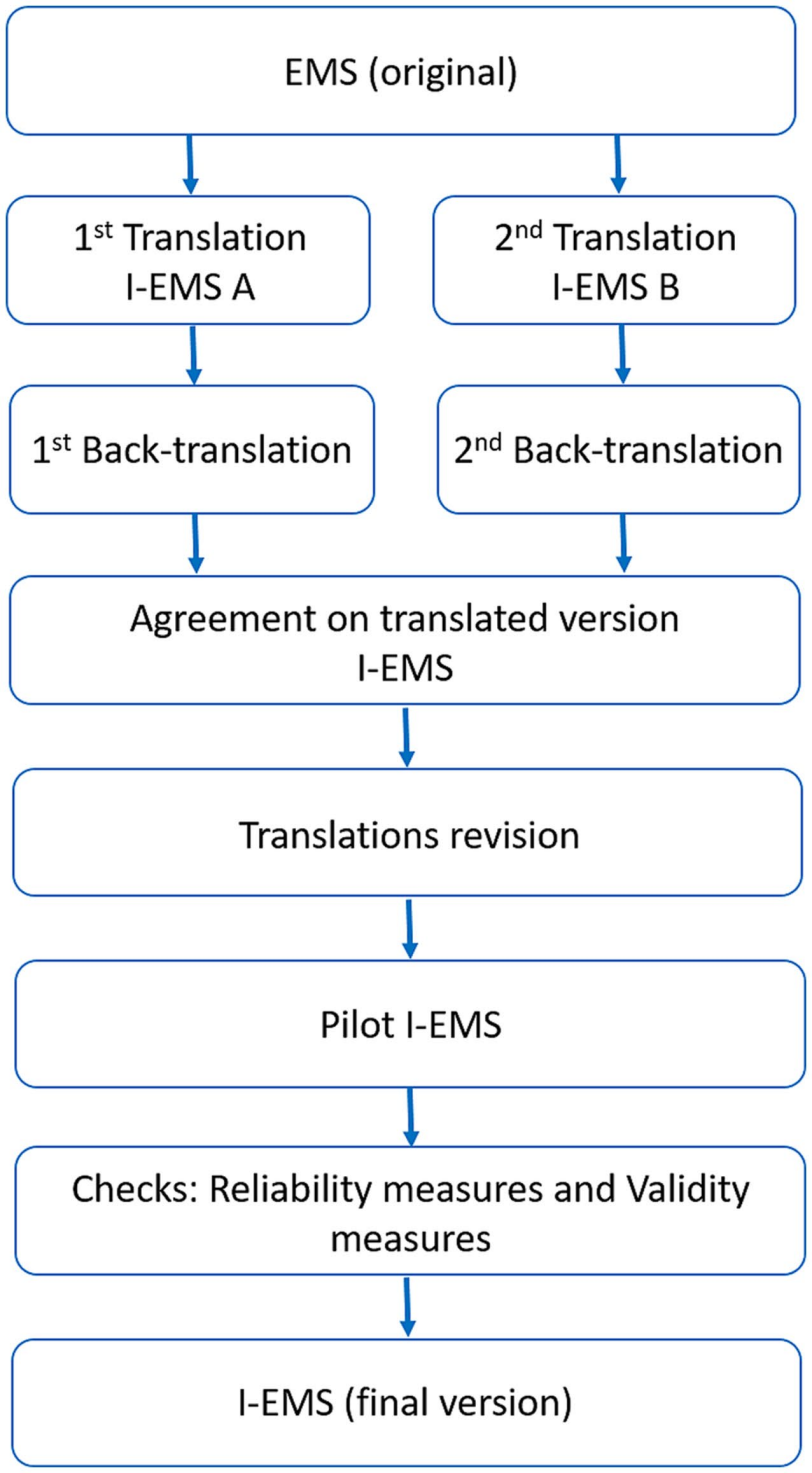
Methodological process that explains the translation and validation of the scale from the original version to the Italian version. Note that the final translated version of the scale was obtained following a double translation and comparison between them. A subsequent revision and check on reliability and validity were performed.

The translation phase followed the process depicted in Figure 1, according to Cosmin checklist (17). In particular, two different teams were formed in order to translate the Version 2 of EMS (12, 13) into Italian. Each team included two bilingual professionals working in the rehabilitation sector (physiotherapists). The obtained translations were sent to two native English speakers who independently back translated the two versions. In the next step, a committee composed of two physiotherapists and one physician reviewed the outcomes and concluded on the first version of the I-EMS (Appendix A in Supplementary material). This version was then pilot-test in a group of 10 experienced physiotherapists who were asked to give their opinion on the clarity and comprehension of each item. For each item, physiotherapists had to answer the following question: ‘Is the item clear and understandable?’ with “Yes,” “No,” “Quite clear/understandable.” The percentages of responses were calculated for each item and the item was possibly corrected if >20% of participants judged it “not clear” or “quite clear/understandable.” After the pilot test, the final version of the I-EMS was produced for phase 2 of the study.

### 2.2. Phase 2: field test and evaluation of I-EMS

#### 2.2.1. Subjects

Patients with different health problems and disabilities admitted consecutively to any hospital ward of IRCCS INRCA (Ancona, Italy) from September 2022 to April 2023 were enrolled in the study, provided they met the following inclusion criteria: hospital admission because of multiple medical issues, 65 years of age or older, medical request for physiotherapy assessment and willingness to participate with signed informed consent. Patients with clinical conditions, reported by the medical personnel, that prevented them from performing the motor tasks required during the evaluation were excluded.

#### 2.2.2. Testing procedure

The participants were simultaneously assessed with the I-EMS by two physiotherapists (rater A and rater B), who were blind to each other’s evaluations. The two raters were randomly chosen between a group of seven physiotherapists. All patients were also assessed using the Italian version of the Barthel Index (BI) (18), that was administered by the health care professional in charge of the patient. All patients were tested between 10.00 am and 2.00 pm, and the time spent on testing with the I-EMS was approximately 15 min. Both assessment tools were used in accordance with the respective manuals. The equipment used in the study was a bed in neutral position, a chair, measuring tape, a stopwatch, and the patients’ walking aids.

#### 2.2.3. Elderly mobile scale

The EMS is a commonly applied mobility outcome measure in the acute hospital setting and consists of 7 items and is scored from 0 to 20 (4). It is administered by observation of physical performance. The EMS items consist of: lying to sitting, sitting to lying, sit to stand, stand, gait, timed walk and functional reach. Four items have four response options (scored 0, 1, 2 or 3) and three items have three response options (scored 0, 1 or 2 for two items and 0, 2 or 4 for one item). Activities are completed with the appropriate level of assistance needed by the patient and a score is given by the rater. Each item scores discrete integer values attributed according to the patient’s ability to perform or not to perform the task and the level of assistance needed.

#### 2.2.4. Barthel index

The Italian version of the Barthel Index (BI) (18) was chosen as the gold standard for determining construct validity (hypothesis testing). The BI is an ordinal scale used to measure performance in activities of daily living (ADL), scoring from 0 (totally dependent) to 100 (totally independent). Ten variables describing ADL and mobility are scored; a higher number corresponds to a greater independence. Each item is scored according to the level of physical assistance needed to perform the required task.

#### 2.2.5. Statistical analysis

Having a small sample size, we preliminarily checked the normality of the distribution of variables by means of the Shapiro–Wilk test in order to choose appropriate statistics. The test showed evidence of non-normality (*p* < 0.0005), so non-parametric tests were used. Descriptive data were presented as mean and standard deviation, median and interquartile range or numbers and percentage, as appropriate. The Mann Whitney *U* test was used to evaluate differences between the total I-EMS score and between each item scores, when administrated by different raters.

To determine interrater reliability, one-way random ANOVA single measures Intraclass Correlation Coefficient (ICC1,1) for the total score, and percentages of agreement and weighted kappa for each I-EMS item were calculated. Moreover, in order to estimate the absolute reliability or measurement error of Measurement (SEM) was calculated for the total I-EMS score, according Formula (1) (19).

(1) $$SEM=SD\left(IEMS_{RATER_{A}},IEMS_{RATER_{B}}\right)*\sqrt{1-ICC}$$

From the SEM, the Minimal Detectable Change with 95% confidence (MDC95) was calculated according Formula (2).

(2) $$MDC95=SEM*1.96*\sqrt{2}$$

As a second step, we assessed construct validity (hypothesis testing). This type of validity examines whether two measures or scales, designed to gauge constructs that are expected to be correlated, actually show correlation. Pearson one-tailed correlation was used to verify the association between I-EMS scale and BI. In fact, if a specific direction of the correlation is predicted, then a one-tailed test can be adopted. If it is known *a priori* that there will be a positive correlation between the two scales, then a one-tailed test, in which the alternative hypothesis states that the correlation coefficient is greater than zero, can be adopted. Correlation coefficient, out coming Pearson one-tailed correlation test, identifies the degree of correlation of I-EMS and Barthel Index. The null hypothesis can be rejected when value of p is less than significance level 0.05.

## 3. Results

The results regarding the evaluation of the clarity of the translation carried out by 10 experienced physiotherapists were good, showing agreement among the physiotherapists interviewed in all the items evaluated, emphasizing the adequacy of the translation, as shown in Table 1.

**Table 1:** Evaluation of the clarity of the translation.

EMS Item	Yes (n, %)	No (n, %)	Quite clear/understandable (n, %)
1. Lying to sitting	8, 80%	1, 10%	1, 10%
2. Sitting to lying	9, 90%	1, 10%	0, 0%
3. Sitting to standing	9, 90%	1, 10%	0, 0%
4. Standing	8, 80%	1, 10%	1, 10%
5. Gait	9, 90%	0, 0%	1, 10%
6. Timed Walk	9, 90%	0, 0%	1, 10%
7. Functional reach	9, 90%	1, 10%	0, 0%

The study on the metric properties of the I-EMS included 61 patients in acute care setting, 26 men and 35 women. The participants’ characteristics are presented in Table 2.

**Table 2:** Demographic characteristics of the study population.

	Total *n* = 61	Male *n* = 26	Female *n* = 35
Age, mean ± std	82.2 ± 8.1	81.4 ± 9.9	82.8 ± 6.5
Marital status, n (%)			
Married	24 (39.4%)	14 (58.3%)	10 (41.7%)
Cohabitant	1 (1.6%)	1 (100%)	0 (0%)
Separated	3 (4.9%)	3 (100%)	0 (0%)
Single	4 (6.5%)	3 (75%)	1 (15%)
Widowed	29 (47.6%)	6 (20.7%)	23 (79.3%)
Educational level, n (%)			
No education	3 (4.9%)	0 (0%)	3 (100%)
Primary education	36 (59%)	15 (41.6%)	21 (58.4%)
Secondary education	16 (26.2%)	7 (43.7%)	9 (56.3%)
University or more	6 (9.9%)	4 (66.6%)	2 (33.4%)
Employment status, n (%)			
Unemployed	1 (1.6%)	1 (100%)	0 (0%)
Retired	60 (98.4%)	25 (41.6%)	35 (58.4%)
Inpatient ward, n (%)			
Cardiology	11 (18.1%)	5 (45.4%)	6 (54.6%)
Geriatrics	9 (14.8%)	5 (55.5%)	4 (44.5%)
Neurology	1 (1.6%)	1 (100%)	0 (0%)
Internal Medicine	7 (11.5%)	1 (14.3%)	6 (85.7%)
Post-acute Care	1 (1.6%)	1 (100%)	0 (0%)
General Surgery	2 (3.2%)	2 (100%)	0 (0%)
Rehabilitation	29 (47.6%)	11 (37.9%)	18 (62.1%)
Pneumology	1 (1.6%)	0 (0%)	1 (100%)

std = standard deviation, n = number of subjects.

Table 3 shows descriptive values of each item and total score (median and interquartile range, and mean and DS, respectively) of I-EMS administered by the two raters (I-EMS-RATER-A and I-EMS-RATER-B) and the result of statistical comparison (Mann Whitney U test) of their scores. The maximum I-EMS score is 20. The average score of the total I-EMS was 14.36 (± 5.00) for the scale administered by the first physiotherapist and 14.41 (± 4.96) for the scale administered by the second one. The average for the total BI was 64.01 (± 23.05). Statistical analysis revealed no significant differences between the scores of individual items and the total score assigned by different raters (Table 3). The ICC for total I-EMS was 0.951 (95% CI 0.949–0.998), SEM was 1.10 and MDC_95_ was 3.06.

**Table 3:** Median and interquartile range of each item of I-EMS-RATER-A and B and mean ± standard deviation of total score obtained among different raters.

EMS item	I-EMS-RATER-A	I-EMS-RATER-B	*p*
1. Lying to sitting (median ± IQ)	1 (0)	1 (0)	1
2. Sitting to lying (median ± IQ)	1 (0)	1 (0)	0.61
3. Sitting to standing (median ± IQ)	1 (1)	1 (1)	0.90
4. Standing (median ± IQ)	1 (0)	1 (0)	1
5. Gait (median ± IQ)	2 (2)	2 (2)	0.68
6. Timed Walk (median ± IQ)	1 (1)	1 (1)	0.75
7. Functional reach (median ± IQ)	2 (1)	2 (1)	0.73
Total score (mean ± SD)	14.36 ± 5.00	14.41 ± 4.96	0.94

Between groups comparisons are reported for each item (*p* < 0.005) after Mann–Whitney test was performed.

I-EMS-RATER-A = scale administration by the first physiotherapist; I-EMS-RATER-B = scale administration by the second physiotherapist.

The absolute agreement between raters and weighted kappa for each item are shown in Table 4. The average absolute agreement across all items was 92.8%, ranging from the 80.32% (item “Gait”) to 100% (item “Standing.” Moreover, weighted kappa showed high values, ranging from 0.822 (“Gait”) to 1 (“Standing”). Values greater than 0.8 indicates optimal agreement between scores obtained by different observers.

**Table 4:** Reliability of each I-EMS items.

I-EMS item	Absolute agreement (%)	Weighted Kappa
1. Lying to sitting	96.72	0.924
2. Sitting to lying	96.76	0.856
3. Sitting to standing	85.24	0.837
4. Standing	100.00	1.000
5. Gait	80.32	0.822
6. Timed walk	98.36	0.902
7. Functional reach	95.08	0.871

Table 5 reports the percentage of absolute agreement among different raters for each item of the scale according the functional ability of the patients. Bottom percentile (< 0.33) encompasses subjects whose global EMS score was less than 10, which stands for completely dependent, upper percentile (>0.66) encompasses patients whose global EMS score is higher than 14, indicating that patient is autonomous. Intermediate percentile (0.33–0.66) is composed by those patients with a borderline trend in terms of mobility independence.

**Table 5:** Percentage of absolute agreement among different raters for each item of the scale according the functional ability.

	Interrater percentile		
EMS item	< 0.33	0.33–0.66	> 0.66
1. Lying to sitting	90	90.90	100
2. Sitting to lying	100	90.90	97.43
3. Sitting to standing	60	81.80	92.30
4. Standing	100	100	100
5. Gait	60	81.80	84.61
6. Timed Walk	100	90.90	100
7. Functional reach	90	100	94.87

**Figure 2:**
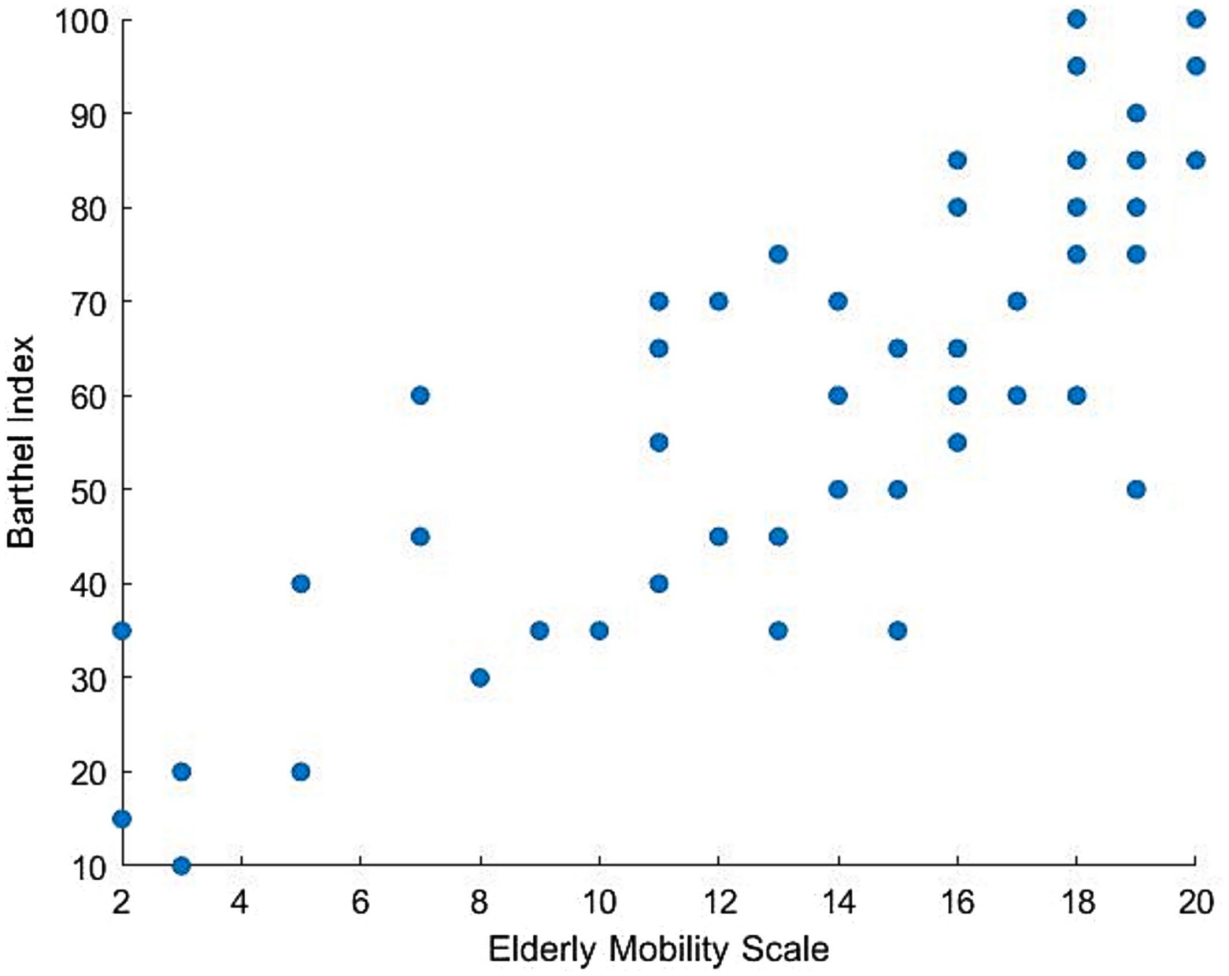
Scatter plot of the distribution of values of EMS-RATER-A and Barthel Index.

The EMS demonstrated construct validity, with a Pearson correlation coefficient between the total I-EMS-RATER-A and the BI scale of 0.827 (*p* < 0.05) and the total I-EMS-RATER-B and the BI scale of 0.834 (*p* < 0.05). Scatter plot of the distribution of values of EMS-RATER-A and BI is shown in Figure 2.

## 4. Discussion

The aim of this study was to develop an Italian version of the Elderly Mobility Scale (I-EMS) and evaluate its construct validity (hypothesis testing) and inter-rater reliability for use with geriatric in-patients. The data showed that the I-EMS has excellent reliability and can be used to accurately measure individual performance. In fact, according to the criteria suggested by Fitzpatrick et al. (20) reliability coefficients of at least 0.90 are required for this purpose, while coefficients >0.70 are acceptable when studying groups. The validity of the I-EMS is supported by the findings of a significant correlation with the functional level as measured by the BI.

As regards inter-rater reliability, the data of the present study are similar or better than the data found for the original version of the scale (16) and for the versions translated in other languages (11, 12). However, the sample enrolled in the present study is about double or more. For the Norvegian version of the EMS (10) only the intra-rater reliability has been studied, so comparisons with the present study are not possible.

Prosser and Canby (16) examined the inter-rater reliability of the original English version of the EMS in 19 patients admitted to a general hospital and found a Spearman’s coefficient = 0.88. The authors did not calculate the error of the measurement, but from the data presented we can argue that the error was very high, because the difference between the score assigned by the two raters was up to 8 points. We computed from these data the Limits of Agreement (LoA) using the Bland–Altman method (21) and found that the mean difference was 1,26, the upper limit 7.4 and the lower limit 4.8. The LoA are an alternative method to compute the maximal random variability in the scores that is expected in 95% of assessments, so they approximate to the MDC95. The metric properties of the original EMS have been also investigated in two other studies (4, 7) that do not report adequate reliability indexes. Kuys and Brauer (22) studied the reliability of a modified English version of the EMS, where one further item was added (stairs climbing) and where the path length for the item “Timed walk” was changed from 6 to 10 meters. The authors found excellent inter-rater reliability (ICC = 0.933) but their results are hardly comparable with the published literature and with the present study, since in the modified EMS the total score was altered to 23 points.

Our findings are very similar to what found by Cuijpers et al. (12) for the Dutch version of the EMS. In that study, two raters administered the Dutch EMS in a sample of 28 geriatric patients on two occasion, and the comparison of their assessment showed excellent reliability (ICC = 0.95 and 0.96 on the first and on the second occasion, respectively). The authors explored the error of the measurement using the Bland–Altman approach, finding a value of 3 points, i.e., just the same that we found in the present study.

Slightly better findings have been reported for the Swedish version of the EMS (11), that was administered twice (before and after midday) in a sample of 30 subacute stroke patients at both admission and discharge. The analyses showed ICC = 0.98 and 0.99, respectively. The authors did not report the error of measurement but provided the necessary data for computation. From the data presented in the article, we estimated that the MDC95 for admission and discharge assessments were 2.63 and 1.69, respectively. These values are lower than the values found in the present articles, but several features might explain the difference. First, although the authors state that they aimed at investigating the inter-rater reliability of the scale, actually only 21 out of 30 participants were assessed by different raters, whereas 9 subjects were assessed by the same rater both before and after midday. Thus, the authors studied the test–retest reliability of the EMS mixing inter-and intra-rater reliability, a procedure that likely enhanced the agreement between the two assessments. Moreover, at discharge half of participants reached the maximum EMS score (=20), and this fact also likely led to overestimate reliability. Last, the authors do not state clearly which type on ICC was computed, and in particular whether the reported values are the ICC for single measures or the much higher ICC for average measures. We also computed the Bland–Altman LoA from their data about admission assessment, finding a much higher variability of the scores (upper limit: 4.85; lower limit: 3.78), that is not consistent with a MDC95 lesser that 3 points.

Among the quoted literature, only Kuys and Brauer (22) reported ICCs about the inter-rater reliability of individual EMS items, that ranged from 0.933 to 1.0. The average absolute agreement was 97.4%, i.e., slightly higher than the value found in the present study but, as mentioned, the comparison is questionable due to the addition of a new item and the altered path length for gait speed assessment in the modified scale. In the present study kappa values were always higher than 0.80, indicating “almost perfect agreement” (23) among raters. However, two items (“Sitting to standing” and “Gait”) showed higher variations, highlighting the need for further training and standardization to improve agreement across raters.

Kuys and Brauer (22) also examined reliability separately in subgroups of patients according to third percentiles of function, and found that inter-rater reliability increased with increasing level of function, although the difference was not significant. We did not observe a similar trend, likely because of different level of function in the three groups: our participants in the bottom and in the top percentiles had a I-EMS total score < 10 and > 14, respectively, compared to <16 and > 21 in the study of Kuys and Brauer (22).

Due to the lack of a validated Italian gold standard to measure mobility, the construct validity of the I-EMS was estimated in the present study by correlating the scale with the BI, which was chosen as the reference standard also by other authors (4, 16). These studies adopted the modified version of the Barthel Index suggested by Collins et al. (24), which retains the different levels of performance for each item and differs from the original one only in the scoring system (1-point instead of 5-points increments between the different levels of performance). Therefore, the two versions are equivalent (the original BI score can be transformed into the modified score by simply dividing it by 5) and the different score does not affect the correlations found with other scales. The BI is not strictly a measure of mobility, but rather a more comprehensive measure of functional capacity that assesses independence in all basic activities of daily living (ADLs) and includes some aspects of mobility. However, mobility, i.e., “the ability to move oneself…within environments that expand from one’s home to the neighborhoods and to regions beyond” (25), impacts on most basic ADLs and self-care, Therefore, the two scales (I-EMS and BI) measure different constructs but should be related, and the finding of a positive correlation between them confirms the construct validity of the I-EMS. Our results are in agreement and strengthens the findings of previous studies (4, 16). In Smith (4) the Spearman’s correlation between EMS and BI in a sample of 36 older patients was 0.962, in Prosser et al. (16) it was 0.787 in a sample of 66 patients. The study on the modified version of the EMS (21) found a similar correlation (rho = 0.725) with the motor section of the Functional Independence Measure, that is the other instrument largely used to assess independence in basic ADLs.

The implications of the findings are significant for clinical practice and research in the field of geriatric rehabilitation. The I-EMS provides a reliable and valid tool to specifically assess mobility in older in-patients. Its ease of use and relatively short administration time make it suitable for routine clinical assessment. The availability of an Italian version of the scale fills a gap in the existing literature and allows for standardized assessment in Italian-speaking populations.

However, a limitation of this paper is the large number of patients who come from the rehabilitation ward and who could therefore be better prepared from a functional point of view. Furthermore, it would be interesting to investigate the other psychometric characteristics of the scale.

## 5. Conclusion

The I-EMS provides a valuable tool to assess the mobility and motor function of frail older patients in acute care settings. Its ease of administration, short evaluation time, and lack of specific training requirements make it a practical choice for healthcare professionals. Furthermore, it can safely be used in geriatric hospital care in order to carry out a comprehensive geriatric assessment and a functional analysis of the patient’s own.

Finally, I-EMS allows to evaluate functional mobility as a single tool, while the same motor skills are generally assessed with several rating scales, such as Trunk Control Test, Standing Balance, Gait Speed Test (4 meters) and Functional Reach.

## Data availability statement

The raw data supporting the conclusions of this article will be made available by the authors, without undue reservation.

## Ethics statement

The studies involving humans were approved by Ethic Committee of the IRCCS INRCA Hospital (Ancona, Italy). The studies were conducted in accordance with the local legislation and institutional requirements. The participants provided their written informed consent to participate in this study.

## Author contributions

MN: Conceptualization, Writing – review & editing. EC: Conceptualization, Data curation, Investigation, Writing – original draft. EB: Investigation, Writing – original draft. LD: Investigation, Writing – original draft. CP: Investigation, Writing – original draft. FM: Investigation, Writing – original draft. PF: Investigation, Writing – original draft. SL: Investigation, Writing – original draft. RB: Writing – review & editing. FB: Data curation, Formal analysis, Methodology, Writing – original draft. EM: Methodology, Writing – original draft. MB: Writing – review & editing. IB: Writing – original draft. GR: Writing – review & editing.
