# Supplementary material for: Evaluation of the Italian version of the elderly mobility scale in older hospitalized patients
Source: Front Public Health. 2023 Nov 15;11:1274047. doi: 10.3389/fpubh.2023.1274047 (PMC10684775; doi:10.3389/fpubh.2023.1274047)
Supplement: Supplementary file 1 [file Data_Sheet_1.PDF]

EMS - I

Nome del paziente____

**COMPITO**	**Data**			
Da disteso a seduto	**2** Indipendente<br>**1** Necessita di aiuto di 1 persona<br>**0** Necessita di aiuto di 2 o più persone			
Da seduto a disteso	**2** Indipendente<br>**1** Necessita di aiuto di 1 persona<br>**0** Necessita di aiuto di 2 o più persone			
Da seduto a in piedi	**3** Indipendente (meno di 3 secondi)<br>**2** Indipendente (più di 3 secondi)<br>**1** Necessita di aiuto di 1 persona<br>**0** Necessita di aiuto di 2 o più persone			
In piedi	**3** In piedi senza appoggio e capace di raggiungere un oggetto<br>**2** In piedi senza appoggio ma necessario sostegno per raggiungere un oggetto<br>**1** In piedi ma appoggio necessario<br>**0** In piedi solo con sostegno fisico di un'altra persona			
Cammino	**3** Indipendente (+/- bastone)<br>**2** Indipendente con deambulatore<br>**1** Capace di spostarsi con un ausilio ma in maniera irregolare/insicura<br>**0** Necessita di aiuto fisico per camminare o di supervisione costante			
Misurazione del tempo del cammino (percorrere 6 metri)	**3** Meno di 15 secondi<br>**2** 16-30 secondi<br>**1** Più di 30 secondi<br>**0** Incapace di percorrere 6 metri			
	Tempo registrato (secondi)			
Functional Reach	**4** Più di 20 cm<br>**2** 10-20 cm<br>**0** Meno di 10 cm			
	cm effettivi			
	**PUNTEGGI**	**/20**	**/20**	**/20**
	**Iniziali Valutatore**			

Punteggio inferiore a 10 – generalmente questi pazienti sono **<u>dipendenti</u>** nelle attività di movimento; richiedono aiuto nelle ADL di base, come i trasferimenti, andare in bagno e vestirsi.

Punteggio tra 10 e 13 - generalmente questi pazienti sono **<u>borderline</u>** in termini di sicurezza nella mobilità e di indipendenza nelle ADL ad esempio necessitano di una forma di aiuto nelle attività di mobilità.

Punteggio superiore a 14 - generalmente questi pazienti riescono a svolgere attività di mobilità da soli ed in sicurezza e sono **<u>indipendenti</u>** nelle ADL di base.
